# Light

**Published:** 1889-11-30

**Authors:** 


### Words of Consolation.

### "LIGHT."

*(For Reading to the Sick.)*

We have peeped through the gates of paradise, set ajar for us by the dear Lord Himself, and tried to realise some of the glories which the blessed enjoy in those gardens of the great King. We will now see of whom this happy company consists, which is ever rejoicing in the Lord before the throne. There are the pure in heart, for it is promised that they shall see God; the poor in spirit, for theirs is the kingdom of heaven; the mourners, for they shall be comforted; the faithful, who have fought a good fight and endured manfully to the end, for they shall be saved. All these

Loyal hearts and true
Stand ever in the light,
All rapture through and through,
In God's most holy sight.

And cannot we imitate this purity, this meekness, this valiant fighting for the truth, and so prepare ourselves also to live ever with the Lord? We are told that in the heavenly Jerusalem there is no need of the sun, neither of the moon to shine in it: for the glory of God did lighten it, and the Lamb is the light thereof. Let us set our faces heavenwards, and bask in the rays of the Sun of Righteousness which stream alike over His Church above and His Church on earth. Alas! at first when we do this we are filled with despair. We see in its pure brightness our selfishness, our vanity, our mean aims, our paltry jealousies, our sinful lusts, stand out in all their ugly blackness. But as our Saviour shines in the holy city for the strength of His saints, so on earth He cleanses, enlightens, warms, and comforts his poor, wretched, penitent children. He comes to us with healing on His wings, He is the light to lighten the Gentiles, and to be the glory of His people Israel. He is the Light of the World, and those who follow Him shall not walk in darkness, but shall have the light of life. With such promises given us, are we carefully striving to follow in our Lord's footsteps? Unhappily, too many shun the glare of truth upon their path; they love darkness because their deeds are evil. Some really love the light, but hate the trouble of seeking for it. Others are ignorant. As men in a cave with their backs to the light can see nothing real, only the distorted shadows of the objects which pass behind them, cast on the wall in front, so these poor souls are content not to know the truth other than as it comes to them in strange doctrines, in grotesque forms of so-called worship; they will not, perhaps cannot, turn to the light, and so lose the blessing which might be theirs. But for those happy souls who have set their faces Zionward and look steadfastly on

Those dear tokens of Christ's passion,
Which His dazzling body bears,

Paradise has already begun on earth. Like the eagle, they soar ever upward on the wings of faith, gazing on their Sun of Righteousness without being blinded; or, as flowers standing in the noonday heat become more brilliant in colour, more luxuriant in growth, so they flourish.

Faith is their fixed, unswerving root,
Hope their unfading flower,
Fair deeds of charity their fruit.

Their perfection will be in the glorious bowers of paradise.

